# An Incomplete Medical Record: Transfer of Care From Emergency Medical Services to the Emergency Department

**DOI:** 10.7759/cureus.22446

**Published:** 2022-02-21

**Authors:** Jeffrey S Lubin, Akash Shah

**Affiliations:** 1 Department of Emergency Medicine, Penn State Health Milton S. Hershey Medical Center, Hershey, USA; 2 Department of Public Health Sciences, Penn State College of Medicine, Hershey, USA; 3 Department of Obstetrics and Gynecology, Penn State Health Milton S. Hershey Medical Center, Hershey, USA

**Keywords:** emergency department, emr, transfer of care, ems, prehospital

## Abstract

Background: Transition of care from Emergency Medical Services (EMS) to the Emergency Department (ED) represents an intersection at high risk for error. Minimal research has quantitatively examined data transfer at this point. In Pennsylvania, this handoff consists of a transfer-of-care form (TOC) provided by EMS to ED in addition to a verbal report. A prehospital patient care report (PCR) is later filed by EMS up to 72 hours after concluding care.

Objective: To evaluate the congruence between prehospital records provided at handoff and the final PCR found in the patient’s medical record. Our hypothesis was that there would be discrepancies between the TOC and final PCR.

Methods: A retrospective chart review was conducted comparing the TOC from a single EMS agency to the final PCR found in the electronic medical record. A convenience sample of 200 patients who received advanced life support transport over a one-month period were included. Metrics to assess the discrepancy between the reports included chief complaint, allergies, medications, systolic and diastolic blood pressure (SBP and DBP), pulse, respiratory rate (RR), Glasgow Coma Score (GCS), and prehospital treatment provided. The level of agreement between the two sources was compared using kappa statistics and concordance correlation coefficients (CCC) with 95% confidence intervals.

Results: Of the 200 encounters that met inclusion criteria, 72% had matching chief complaints between the TOC and PCR. Medications matched in 66% and allergies matched in 82%. Up to three BP, pulse, and RR readings were collected; only 30% of the third BP readings were available from the TOC, while 68% were available from the PCR. Comparing the three SBP values on the TOC to respective counterparts on the PCR showed a substantial correlation (all CCC >0.95). Pulse and DBP values had moderate-to-substantial correlation (CCC: 0.93, 0.94, 0.96 and 0.77, 0.92, 0.94 respectively). RR showed inconsistent correlation (CCC: 0.37, 0.84, 0.94). GCS showed a moderate correlation between the two forms (CCC: 0.81).

Conclusion: There were significant differences between the information transferred to the ED through the TOC compared to what was recorded in the PCR. Further evaluation of the TOC process is needed to improve accuracy.

## Introduction

Transition of care from one provider to the next is a fragile point in healthcare during which there is a high risk for medical error that could negatively impact a patient’s course of treatment. Patient hand-offs specifically between prehospital Emergency Medical Services (EMS) and the Emergency Department (ED) may be even higher-risk events that can threaten a patient’s safety because care decisions often need to be made quickly and without full documentation of the care provided by EMS [1]. A review of the literature shows that the transition of care between EMS and ED is an area of concern, however, existing research in the field is sparse [2-5]. A systematic review of the literature has shown that several transition guidelines have yielded mixed results in streamlining the transition at this intersection [6-9]. However, both EMS and ED personnel believe that this handoff represents a dynamic point in emergency medical care that can have significant implications on a patient’s course of treatment. [10,11].

In Pennsylvania, EMS to ED handoff consists of a standardized transfer-of-care form (TOC) that is provided by EMS in addition to a verbal report. The final prehospital patient care report (PCR) must be filed within 72 hours after EMS concludes care. The PCR is eventually entered into the patient’s hospital record. Due to the disjointed process of filing these reports, we hypothesized there would be discrepancies between the TOC and the final PCR submitted by EMS. This article was previously presented as a meeting abstract at the National Association of EMS Physicians Annual Meeting in January 2019.

## Materials and methods

We conducted a retrospective chart review of a convenience sample of patients treated at our medical center after being transported by a single EMS agency and receiving advanced life support (ALS) care during May 2017. The EMS system covers three counties of our state, primarily in suburban areas. Data was extracted from a third party, web-based platform that houses all of the agency’s prehospital records (emsCharts.com). Patients outside the timeframe, those who did not receive ALS treatments, or those brought by a different EMS agency were excluded from the study. Patients were also excluded if no TOC was present in their chart. One of the study’s co-authors reviewed all records for metrics used to assess the congruence between the two forms, which included chief complaint, allergies, medications, systolic and diastolic blood pressure (SBP and DBP), pulse, respiratory rate (RR), Glasgow Coma Score (GCS), and prehospital treatment provided. Each vital sign was recorded up to three times. Prehospital treatment provided included use of oxygen, IV placement, blood sugar levels, labs, medication, use of continuous positive airway pressure (CPAP), defibrillation, cardioversion, transcutaneous pacing, splinting, immobilization of cervical spine, or establishing an airway.

The level of agreement between the categorical information that was collected on the TOC and that included in the PCR was estimated by kappa statistics and reported with corresponding 95% confidence intervals. The comparison of any continuous variables collected on the TOC versus PCR was evaluated for agreement by concordance correlation coefficients (CCC) and corresponding 95% confidence intervals, as well as paired t-tests to compare the average differences versus zero. Our organization’s Institutional Review Board reviewed and approved this study.

## Results

In May 2017, the total number of patients receiving ALS treatment by the participating EMS agency and were transported to Hershey Medical Center ED was 342. Of those, 142 were excluded due to a lack of a TOC in the chart. Of the remaining 200 patients, 102 (51%) were male. The mean age was 62.3 years. Of the metrics used to assess congruence between the two forms, chief complaints were present and matching in 144 (72%) cases. Patient medications were present and matching in 132 (66%) cases. Allergies matched in 164/200 (82%) cases.

Vital signs, inclusive of SBP, DBP, pulse, and RR were recorded up to three times. The number of entries for blood pressure, pulse, and RR values in each of the forms is shown in Table 1. SBPs showed a strong correlation in all three recordings with CCC > 0.95. DBP values showed moderate correlation with CCC values of 0.77, 0.92, and 0.94, in each of the three values, respectively. Pulse values showed a strong correlation between the two forms (CCC: 0.92, 0.94, 0.96). RR showed inconsistent correlation between the two forms (CCC: 0.37, 0.84, 0.94). Of the 200 charts reviewed, 182 GCS values were present on the TOC and 197 were present on the PCR. Of the values present, there was a moderate correlation between the two reports (CCC: 0.81).

**Table 1:** Vital Sign Recordings PCR: prehospital care report; TOC: transfer of care; RR: respiratory rate

Number of BP Entries	TOC	PCR
1	165	193
2	119	170
3	60	136
Number of Pulse Entries		
1	168	197
2	117	172
3	61	143
Number of RR Entries		
1	161	193
2	117	164
3	61	125

Kappa statistics were used to assess the congruence in prehospital care provided between the two forms, these values are shown in Table 2. Five of the prehospital care options not listed in the table showed 100% congruence or kappa value of 1. These included defibrillation, cardioversion, transcutaneous pacing, wound care, and splinting.

**Table 2:** Congruence in Prehospital Care Provided IV: intravenous line; CPAP: continuous positive airway pressure; CI: confidence interval

Prehospital Intervention	Kappa Value [95% CI]
Oxygen	0.83 [0.75-0.91]
IV	0.85 [0.76-0.93]
Blood glucose	0.82 [0.73-0.91]
Labs	0.86 [0.79-0.93]
Medication	0.85 [0.76-0.93]
CPAP	0.80 [0.41-1.00]
C-spine Immobilization	0.24 [-0.15-0.63]

## Discussion

A 2013 position statement, reaffirmed in January 2019, on “Transfer of Patient Care Between EMS Providers and Receiving Facilities” was jointly authored by the American College of Emergency Physicians (ACEP), the Emergency Nurses Association (ENA), the National Association of EMS Physicians (NAEMSP), the National Association of Emergency Medical Technicians (NAEMT), and the National Association of State EMS Officials (NASEMSO). It highlights the important points regarding the transfer of care: mutual respect between care team providers, minimum key information and copies of all tests performed by EMS, appropriate TOC documentation, a complete and accurate PCR, and timeliness of that report [12]. Despite this, our study suggests that there is a gap between the documentation provided at the time of TOC compared to the final documentation.

Previous research has been done to investigate this intersection between EMS and the ED. It is well-established that this intersection is an integral point in maintaining quality patient care [1,13]. Much of the work in this area has focused on qualitative collection of provider experiences, showing that providers on either side of the handoff see room for improvement. Focus groups, videographic assessment, and audio recordings of EMS providers have shown that lack of direct communication between providers and lack of a standardized protocol create a difficult environment for a successful transition [10,13,14]. These studies, however, only show the gap in verbal communication. Several other studies have been conducted to discern the barriers to an effective handoff, many of which state that communication between EMS and ED is a major preventable cause of patient harm [4,9,15,16]. Little research has been done to compare the information gathered during an incident to what ultimately ends up in the patient’s medical record.

This study represents one of the first to quantitatively evaluate data transferred between the EMS and the ED. EMS providers are an integral part of a patient’s care and documentation of their care inevitably should become a part of their medical record. Initial comparison between the two forms shows that there were discrepancies between the data provided to the ED on the TOC and the data that was later filed in the PCR. In 41.5% of the transports, the TOC was completely absent. Furthermore, basic identifying information including chief complaint, medications, and allergies did not match consistently. Although chief complaints matched in 72% of cases, the primary reason they matched is because they were missing in both forms in all of these 144 cases. For allergies, of the 164 cases that matched between the two forms, 163 were missing in both forms. With 41.5% of the TOC missing, 72% of all chief complaints missing, and 81.5% of all allergies missing on both forms, it is clear that significant data is missing in the information that is passed on to the ED and what is being charted by EMS.

Comparing vital signs and GCS between the two forms showed that there was varied concordance between blood pressures, with SBPs being strongly concordant, while the DBPs were poor to moderately concordant. As these two values are reported together, this raises the question of how they had different levels of concordance and which of the values reported were accurate. There was also a poor correlation between the two GCS values, which could be an important insight into a patient’s status in transport. Between the two forms, there was also variation in how many times the vital signs were recorded. Overall, less data points were present in the TOC form than what was charted by EMS in the PCR. As shown in Table 1, blood pressure, pulse, and respiratory rates showed similar trends in a number of entries. These vital signs are valuable data points in clinical decision-making and must be provided to ED staff [17,18].

When comparing the prehospital care provided, several of the treatments showed kappa values above 0.8, showing strong agreement between the two forms. One exception to this was the agreement between the two forms about cervical spine stabilization, which was in fair agreement. For treatment such as c-spine immobilization, this information is likely obvious with the arrival of the patient. This is simply an example of how several items were either not present in different forms between the two data sets.

Comparing this study to similar studies examining the transition from EMS to ED, our results yielded similar findings [2,5,8]. This study shows that there are gaps in communication as well as gaps in transfer of information at the intersection of EMS and ED, as was reported in qualitative findings from these studies. These findings highlight an important gap as the information that is lacking directly impacts patient care. Missing report of allergies and medications during the transfer of care could have avoidable life-threatening consequences [19]. Similarly, trends in vital signs can provide important data points that could change or guide management upon arrival to the ED. Lastly, all care provided on the way to the hospital can have implications on the course of treatment patients receive in the hospital [20-22].

We recognize the limitations of this study, which was conducted to illuminate the fragile nature of the transition between EMS and the ED. One significant limitation of this study is that it does not quantify the data that is transferred verbally. This may result in an overestimation of the discordance between the two data sets, however, quantifying the data that is transferred verbally would be a challenging task with limitations of its own. Another limitation is that this study only captures this transition over a one-month period at a single ED with a single EMS agency. This may not have any effect on the data, however, to assess the generalizability of the data set, it is important to further explore this area. Ultimately the data that is transferred from EMS to the ED can have serious implications on health care decisions and pose a risk to the patients being cared for due to inaccuracy of the information and potential duplication of inaccurate information.

## Conclusions

Although patient handoff is a critical and error-prone time in patient care, there is an inconsistency between the information transferred to the ED through the TOC form and what is recorded in the ultimate PCR. The delay between the transfer of care and the creation of the complete prehospital care report creates an opportunity for miscommunication. Valuable data points may be unavailable to the treatment team, resulting in possible errors in patient care. Further evaluation of the TOC process is needed to improve current or establish new practices.
